# Is empiric proton pump inhibition in patients with symptoms of extraesophageal gastroesophageal reflux justified?

**DOI:** 10.1186/s12876-023-02945-7

**Published:** 2023-09-06

**Authors:** Reidar Fossmark, Eivind Ness-Jensen, Øystein Sørdal

**Affiliations:** 1 Department of Clinical and Molecular Medicine, NTNU, Norwegian University of Science and Technology, Prinsesse Kristinas gate 1, Trondheim, 7030 Norway; 2 Medicus Endoscopy, Trondheim, Norway; 3 HUNT Research Center, Department of Public Health and Nursing, NTNU, Norwegian University of Science and Technology, Levanger, Norway; 4 Department of Medicine, Levanger Hospital, Nord-Trøndelag Hospital Trust, Levanger, Norway; 5 Department of Molecular Medicine and Surgery, Karolinska Institutet and Karolinska University Hospital, Stockholm, Sweden

**Keywords:** Gastroesophageal reflux disease, Proton pump inhibitors, Laryngopharyngeal reflux, Impedance

## Abstract

**Background:**

The prevalence of gastroesophageal reflux disease (GERD) has had a marked increase in Western countries with a paralleling interest in extraesophageal (EE) manifestations of GERD, including laryngopharyngeal reflux (LPR). There are considerable differences in clinical practice between gastroenterologists, otolaryngologists and pulmonologists.

**Methods:**

In this narrative review we address some of these controversies concerning EE manifestations of GERD and LPR.

**Results:**

It is disputed whether there is causal relationship between reflux and the numerous symptoms and conditions suggested to be EE manifestations of GERD. Similarly, the pathophysiology is uncertain and there are disagreements concerning diagnostic criteria. Consequently, it is challenging to provide evidence-based treatment recommendations. A significant number of patients are given a trial course with a proton pump inhibitor (PPI) for several months before symptoms are evaluated. In randomized controlled trials (RCTs) and meta-analyses of RCTs PPI treatment does not seem to be advantageous over placebo, and the evidence supporting that patients without verified GERD have any benefit of PPI treatment is negligible. There is a large increase in both over the counter and prescribed PPI use in several countries and a significant proportion of this use is without any symptomatic benefit for the patients. Whereas short-term treatment has few side effects, there is concern about side-effects after long-term use. Although empiric PPI treatment for suspected EE manifestations of GERD instead of prior esophageal 24-hour pH and impedance monitoring is included in several guidelines by various societies, this practice contributes to overtreatment with PPI.

**Conclusion:**

We argue that the current knowledge suggests that diagnostic testing with pH and impedance monitoring rather than empiric PPI treatment should be chosen in a higher proportion of patients presenting with symptoms possibly attributable to EE reflux.

## Gastroesophageal reflux disease and extraesophageal manifestations

The diagnosis of gastroesophageal reflux disease (GERD) is currently defined by the Lyon consensus [1] as well as the ROME IV criteria [2]. GERD is increasing in prevalence in Western countries and the cause is multifactorial. Obesity is a prevalent cause of GERD. The significance of obesity as a risk factor for GERD is illustrated by the finding of esophagitis in more than 50% of patients being candidates for bariatric surgery [3]. The prevalence of Helicobacter pylori (H pylori) infection has declined rapidly. Individuals with H pylori infection and oxyntic mucosal atrophy have gastric hypoacidity that reduce acidic refluxate and therefore protects against GERD including Barrett’s esophagus [4]. The relative importance of the two mentioned phenomenons will most likely vary between populations.

Paralleling the increase in GERD there has been a growing interest in extraesophageal (EE) manifestations of GERD which include laryngitis, cough, hoarseness, dysphonia, asthma, pulmonary fibrosis and pneumonia [5]. About one third of patients with an established diagnosis of GERD have symptoms attributed to EE reflux [6, 7], whereas in patients without verified GERD these symptoms are unspecific of EE reflux since many other conditions and diseases may cause similar symptoms In addition, therapeutic approach varies considerably among gastroenterologists and other specialists involved with this group of patients. In this mini review we will cover some of the crucial elements and controversies within the field of EE manifestations of GERD.

## Pathophysiology and implications for diagnosis and treatment of EE manifestations

Reflux of gastric contents above the upper esophageal sphincter has traditionally been considered the mechanism of airway symptoms in patients with GERD, at least by gastroenterologists. However, many extraesophageal manifestations of reflux are suggested to be triggered by vagal reflexes and that patients with neurologically mediated symptoms do not necessarily have pathological reflux or esophagitis. In experimental studies investigating the effect of esophageal acid exposure on triggering airway hyperresponsiveness, patients with asthma have been divided in their results [8, 9]. However, hypersensitivity as a pathophysiological mechanism is still proposed to explain the association between GERD and airway symptoms by increasing mucus production and lowering the threshold for coughing. Moreover, coughing may induce reflux episodes [10], likely due to the increased intraabdominal pressure initiated by coughing.

Laryngopharyngeal reflux (LPR) is retrograde flow of gastric contents to the larynx and pharynx through the esophagus. The term was coined and preferred by otolaryngologists who defined LPR as “backflow of the stomach contents in to the throat, that is, into the laryngopharynx” [11]. It was stated that LPR differed from classic GERD in pathophysiologic mechanics as well as treatment response [11] which initiated a persistent controversy. LPR may be described as a phenotype of GERD by some gastroenterologists [12] whereas e.g. the American Gastroenterology Association (AGA) expert review on EE GERD published in 2023 does not use the term LPR at all [13]. LPR has received attention among otolaryngologists over the past decades, as it has been claimed to cause a variety of disorders affecting the upper aerodigestive tract, including dysphonia, oropharyngeal dysphagia, globus, and benign laryngeal lesions [13, 14], all commonly encountered by otolaryngologists.

The pathophysiology of LRP has obvious aspects in common with GERD as the pathogenesis of LPR includes that refluxate must travel through the entire esophagus. Indeed, patients with LPR have significantly more acidic reflux than controls [15], but nonacidic reflux episodes reaching the pharynx that were associated with cough seem to be even more prevalent [16] underlining the necessity of proximal impedance measurement. However, it has also been proposed that the epithelium of the larynx and pharynx is more vulnerable to refluxate than the esophagus [17]. Diagnostic testing to find LPR has been complicated by the perception that the disease is caused by a pathologic response to physiologic reflux [18], which makes conventional 24-hour pH and impedance testing problematic due to the perceived low sensitivity of the methods. The reflux episodes of the proximal esophagus and upper esophageal sphincter may be measured, whereas validated cut-off values impedance and pH in the pharynx do not exist [19].

Another topic of controversy is which component of gastric content that is pathogenic in EE GERD. Gastric juice contains hydrochloric acid which may cause tissue damage depending on concentration and exposure time. Furthermore, gastric juice also contains pepsin derived from chief cells in the gastric oxyntic mucosa that has a proteolytic activity at pH below 4 [20]. Pepsin has been measured in the pharynx and proposed as a diagnostic marker of LPR [21, 22], but there are no clinically validated methods for pharyngeal pepsin measurement [23] and the significance of demonstrating pepsin *per se* at an anatomical location with neutral pH may be questioned. Additional components of the gastric contents include bile acids, which may also cause tissue injury [24]. Delineation of the pathogenetic role of acidic versus non-acidic reflux is possible by studying the effect of fundoplication as opposed to proton pump inhibitors (PPI). Despite that laparoscopic Nissen fundoplication effectively eliminated reflux in such studies, there is no convincing data supporting its efficacy in atypical syndromes that are unresponsive to high dose PPI therapy [19, 25]. Specifically, in a controlled trial of patients with LPR unresponsive to PPIs, no improvement was seen in laryngeal symptoms in the subgroup subsequently treated with surgical fundoplication [26].

Uncertainties and controversies over the pathogenic factor in the refluxate, as well as the role of vagally mediation of symptoms without measurable reflux in the pharynx, complicates the interpretation of studies as well as the symptoms of individual patients in the clinic.

## The lack of diagnostic gold standard of EE GERD

Succeeding uncertainties and disagreement concerning the pathophysiology, there is no consensus on how a relationship between suspected EE manifestations and GERD should be established. Nevertheless, diagnostic testing is considered useful as it may rule out the diagnosis of GERD, whereas there is disagreement on what proportion of patients that should be undergo objective testing.

Laryngoscopy findings are essential in the diagnosis of LPR and there has been a strong belief that erythema, edema, hypertrophy and granulation are reliable signs of inflammation caused by LPR. However, the findings at laryngoscopy that have been proposed to be associated with LPR have also been found in the majority (86%) of asymptomatic volunteers [27] and the presence of such findings were unrelated to symptoms, smoking, alcohol or asthma. Aiming to increase the diagnostic yield of laryngoscopy with “disease-specific” instruments measuring the disease severity, the Reflux Finding Score (RFS) [28] has been used during laryngoscopy. Furthermore, a nine item questionnaire Reflux Symptom Index (RSI) [29] has also been used by laryngologists to aid the diagnosis of LPR [28]. However, the diagnostic performance of these scores is poor and the correlation with pH studies and response to PPI treatment is indeed variable [30–33]. Other suggested non-invasive tests such as the salivary pepsin test ( [19, 34, 35], bile acid detection in saliva or mucosal biopsies [36]suffer from insufficient specificity and have not proven useful in a clinically relevant setting.

Esophagogastroscopy is important when establishing a diagnosis of GERD in clinical practice, however, it has a low diagnostic yield in the context of EE manifestations. Whereas the majority of patients with esophagitis may be asymptomatic [37], esophagitis may be seen in 20% of patients with primary EE symptoms [38]. In this context it is essential to note that the majority of patient with esophagitis do not have EE symptoms.

Assessing reflux by catheter-based pH and impedance monitoring has several advantages as it allows monitoring of reflux reaching the proximal esophagus. Objective investigations seem essential as the degree of esophageal acid reflux cannot be predicted from presence or absence of GERD symptoms [38]. The majority of patients with suspected LPR may not have pathologic reflux [39] including those with PPI resistant symptoms [40] and a negative objective investigation at an earlier stage may be useful to patients and doctors [41]. A high proportion of reflux episodes may be non-acidic in patients with LRP and impedance monitoring may be of additional value in a large proportion of the patients with symptoms [42]. As a consequence of the emphasis on detection of proximal reflux, a specialized pharyngeal probe developed to detect pH in liquid and aerosol has been designed [43] (Restech Dx-pH, Houston, TX, USA). However, the specificity is limited by episodes of pH-drop that were unrelated to events recorded by an esophageal pH-impedance probe [44], suggesting that pharyngeal pH may fluctuate independent of reflux. Such alterations have also been reported in patients after gastrectomy [45], questioning the concept and relevance of the technology that was used in the experiments.

Among patients with chronic cough there may also be a subgroup of PPI responders that can be identified by rigorous patient selection that includes such investigations [46]. Consequently, objective investigations preceding empiric PPI treatment may be worthwhile to avoid unnecessary treatment and equally imprecise evaluations at follow-up in a large proportion of patients. However, most algorithms for handling patients with suspected symptoms of EE reflux do not include initial pretreatment esophagogastroscopy, pH and impedance monitoring [1, 5, 19, 47]. Low availability of pH- and impedance monitoring, perhaps especially to otolaryngologists could affect the decision to treat before investigations have been made.

## The rationale for empirical PPI treatment

Due to the low cost and practical convenience of prescribing a PPI trial course against symptoms of suspected EE reflux, this may be chosen instead of initial pretreatment diagnostic investigations. Such strategy may be supported by several guidelines including the recent AGA publication that recommended a PPI trial of 12 weeks [5]. However, initial testing was recommended to be tailored to the patients clinical presentation as well as in patients who fail a PPI trial [5] and experts are divided [19].

Although reflux may cause EE symptoms, it is uncommon for reflux to be the dominant cause in patients not also experiencing typical esophageal symptoms and there is little evidence to support that PPI treatment is better than placebo in this patient group. There is some evidence from an RCTs that rabeprazole 20 mg twice daily was significantly better than placebo in patients selected based on symptoms, videostroboscopic evidence of LPR and RFS [48]. A reduction in RFS and RSI was also reported in patients receiving esomeprazole 20 mg twice daily for three months [49]. One study has found that symptoms of postnasal drainage improved after 8 to 16 weeks of lansoprazole 30 mg twice daily compared to placebo [50]. However, several other studies have not found effects of pantoprazole [51] or rabeprazole 20 mg twice daily [52]. In a randomized clinical trial comparing esomeprazole 40 mg twice daily with placebo did not find any benefits in patients with chronic posterior laryngitis [53]. Notably, this study purposely excluded patients with frequent heartburn. In a meta-analysis containing 480 patients from 10 RCTs there was a minor effect of PPI compared to placebo (RR 1.31, 95%CI 1.03–1.67) and interestingly the preplanned analysis of the effects of PPI treatment without dietary and lifestyle modifications did not reach statistical significance [54]. In patients with chronic cough there are no high-quality studies that support that PPI therapy has a benefit [55]. This further weakens the arguments of an initial PPI trial.

In addition to the imprecision of a PPI trial as a diagnostic test, the potential side effects of PPIs should also be kept in mind [56, 57]. While the absolute risk of side effects caused by a PPI trial is low, long-term use in larger populations may significantly increase gastrointestinal infections, alter the gastrointestinal microbiome and increase the risk of gastric cancer. Rebound acid hypersecretion after cessation of PPI use [58, 59] is especially of concern when treating individuals with poor indication of PPI use as it may lead to long-term use with risks that will necessarily outweigh benefits. Symptomatic rebound hypersecretion is likely to be common after a 12-week PPI trial. After eight weeks of esomeprazole 40 mg once daily 44% of healthy volunteers reported symptoms of gastroesophageal reflux versus 15% in the placebo group [60]. Similarly, after cessation of four weeks of pantoprazole 40 mg once daily as many as 44% versus 9% in the placebo group had dyspeptic symptoms [61]. After long-term PPI use, rebound acid hypersecretion lasts between two and four months [62], which may prevent patients from stopping PPI once started. The use of PPI has almost doubled over the past decade in Norway [63] as well as in other countries [64, 65] and the costs are high. Although many patients have good indications for long-term PPI use that clearly outweighs risks of side effects, the concern is obvious when patients without indication or benefit of PPI use starts life-long acid inhibition [57].

Cost effectiveness of a PPI-trial compared to individualized treatment after diagnostic testing with 24 h-pH and impedance testing is determined by the costs of long-term PPI use and the costs of diagnostic procedures in a particular setting. A PPI trial has low specificity and high placebo response [66] but is considered cost effective compared to investigation in patients with typical symptoms of GERD [67]. However, as acknowledged in the Lyon consensus, patients with atypical symptoms of GERD have much lower response rate to a PPI test than patients with heartburn, thereby diminishing the utility of that approach to diagnosis [1, 47]. It is therefore of concern that empiric PPI treatment is endorsed by society guidelines [68] and that it contributes to an overdiagnosis of EE GERD and long-term overuse of PPI [19]. Finally, despite the lack of cost-effectiveness analyses for most countries there are good reasons not to extrapolate analyses and recommendations of EE GERD treatment from e.g. the United States (US) to countries with lower costs of health services. It is therefore noteworthy that even in the US the cost effectiveness of empiric PPI has been questioned and upfront pH and impedance testing has been advocated [69, 70].

## Conclusions

The management of suspected EE manifestations of GERD including LPR is hampered by uncertainties regarding diagnosis, causality, and treatment efficacy. The benefits of a PPI trial lasting three to six months before 24-hour pH and impedance monitoring seem very low. Although this practice is suggested by society guidelines and considered cost effective in many countries it will lead to long-term PPI use in a high number of patients without any benefit and increased risk of side effects. Diagnostic testing with pH and impedance monitoring rather than empiric PPI treatment should be chosen in a higher proportion of patients presenting with symptoms possibly attributable to EE reflux.

## Data Availability

Not applicable.
